# γδ T cells and the PD-1/PD-L1 axis: a love–hate relationship in the tumor microenvironment

**DOI:** 10.1186/s12967-024-05327-z

**Published:** 2024-06-10

**Authors:** Jian Liu, Min Wu, Yifan Yang, Zixuan Wang, Shan He, Xun Tian, Hui Wang

**Affiliations:** 1 Department of Obstetrics and Gynecology, Tongji Hospital, Tongji Medical College, Huazhong University of Science and Technology, Wuhan, China; 2 Department of Gynecologic Oncology, Women’s Hospital, Zhejiang University School of Medicine, Hangzhou, Zhejiang China; 3 Department of Pathogen Biology, School of Basic Medicine, Tongji Medical College, Huazhong University of Science and Technology, Wuhan, China; 4 Department of Obstetrics and Gynecology, Academician Expert Workstation, The Central Hospital of Wuhan, Tongji Medical College, Huazhong University of Science and Technology, Wuhan, 430014 Hubei China; 5 Cancer Biology Research Center (Key Laboratory of the Ministry of Education), Tongji Hospital, Tongji Medical College, Huazhong University of Science and Technology, Wuhan, China; 6 Department of Gynecologic Oncology, Zhejiang Provincial Key Laboratory of Precision Diagnosis and Therapy for Major Gynecological Diseases, Women’s Hospital, Zhejiang University School of Medicine, Hangzhou, Zhejiang China; 7 Zhejiang Provincial Key Laboratory of Precision Diagnosis and Therapy for Major Gynecological Diseases, Women’s Hospital, Zhejiang University School of Medicine, Hangzhou, Zhejiang China

**Keywords:** γδ T cells, PD-1/PD-L1, Immune checkpoints (ICPs), Tumor microenvironment (TME), Immunosuppressive molecules, Immune checkpoint inhibitors (ICIs), Antitumor immunotherapy

## Abstract

Gamma delta (γδ) T cells demonstrate strong cytotoxicity against diverse cancer cell types in an MHC-independent manner, rendering them promising contenders for cancer therapy. Although amplification and adoptive transfer of γδ T cells are being evaluated in the clinic, their therapeutic efficacy remains unsatisfactory, primarily due to the influence of the immunosuppressive tumor microenvironment (TME). Currently, the utilization of targeted therapeutic antibodies against inhibitory immune checkpoint (ICP) molecules is a viable approach to counteract the immunosuppressive consequences of the TME. Notably, PD-1/PD-L1 checkpoint inhibitors are considered primary treatment options for diverse malignancies, with the objective of preserving the response of αβ T cells. However, γδ T cells also infiltrate various human cancers and are important participants in cancer immunity, thereby influencing patient prognosis. Hence, it is imperative to comprehend the reciprocal impact of the PD-1/PD-L1 axis on γδ T cells. This understanding can serve as a therapeutic foundation for improving γδ T cells adoptive transfer therapy and may offer a novel avenue for future combined immunotherapeutic approaches.

## Background

Γδ T cells in humans are classified into three main subgroups according to the γδ chains of T cell receptors (TCRs) [1]. There are three major γδ T cell subsets classified based on their T cell receptor delta variable (TRDV) genes, which are referred to as Vδ1, Vδ2, and Vδ3 [2] The majority of γδ T cells are in the peripheral circulation comprised of the Vδ2 chain and Vγ9 chain paired population, known as Vγ9Vδ2 T cells. The Vδ1 chain and various Vγ chain paired groups are predominantly found in the skin and mucosa. Vδ3 T cells constitute the majority of non Vδ1 cells and non Vδ2 γδ T cells, which are present in a significant proportion of the liver.

Besides this "innate" programming, the subgroups of γδ T cells retain functional plasticity. In the context of TME, -classification of γδ T based on function rather than TCR phenotype, will describe its functions more objectively. This is immune functions are often switchable, allowing them to exert different effector functions based on the inflammatory background and participating receptors [2, 3]. Studies have also indicated that a subset of γδ T cells, known as γδ T17 cells, can secrete IL-17 within the TME [4]. These cells have been proven to drive cancer progression by exerting downstream effects on cancer cells, endothelial cells, and other immune cell populations [5].

Similar to those of αβ T cells, the effector functions of γδ T cells can be differentiated based on the expression of CD45RA and CD27, including naïve (T_naïve_), central memory (T_CM_), effector memory (T_EM_), and terminally differentiated (T_EMRA_) subsets [6]. γδ T cells can directly recognize tumor cells via their TCRs and natural killer cell receptors (NKRs). They are capable of producing interferon γ (IFN-γ) and tumor necrosis factor (TNF), as well as exerting antigen presentation functions to activate αβ T cells, thereby inducing antitumor immunity. Furthermore, when exposed to tumor specific antibodies, γδ T cells can effectively target tumor cells through antibody dependent cellular cytotoxicity (ADCC) [7].

Due to the potent antitumor abilities of γδ T cells, both in vivo and in vitro studies have been conducted to expand these cells, with the aim of utilizing adoptive immunotherapy [8]. γδ T cells exhibit reduced infiltration and frequently experience immune tolerance and exhaustion in the immune microenvironment [9–11]. The swift development of tolerance in γδ T cells has even been observed in terminal cancer patients with B cell tumors [12]. Therefore, gaining a deeper comprehension of the expression and regulation of immunosuppressive molecules in γδ T cells has significant implications for advancing immune checkpoint blockade (ICB) therapy and adoptive transfer therapy. This article provides a comprehensive overview of the involvement of the PD-1/PD-L1 signaling pathway in the proliferation, activation, and antitumor properties of γδ T cells, along with their potential application in immunotherapy.

## The relationship between the expression of immunosuppressive molecules and function of γδ T cells

### The expression of PD-1 and function of γδ T cells in healthy individuals

Vδ2 chains are predominant in peripheral blood (PB), and the proportion of PD-1 expressing Vδ2 T cells decreases with maturity, with the highest expression in healthy umbilical cord blood, followed by Vδ2 T cells from infants and then lowest in adults [13]. After in vitro activation, the expression time of PD-1 in Vδ2 T cells from neonates is longer than that in adult Vδ2 T cells, suggesting that PD-1 may be a crucial regulator in early life [13].

The expression of CD56 and PD-1 can be used to evaluate the cytotoxic potential of Vδ2 T cells in umbilical cord blood. The PD-1^−^CD56^+^ subgroup has higher expression of perforin (PRF1) and higher expression of genes linked with NK-mediated cytotoxicity [14]. Compared to umbilical cord blood cells, peripheral Vδ2 T cells from 12 month-old infants exhibit stronger proliferative responses and cytotoxic functions that are similar to those in adults. The enhanced capacity of Vδ2 T cells in infants to generate cytotoxic mediators is also congruent with a reduction in PD-1 expression and an increase in NKG2A expression [13]. The percentage of Vδ1 T cells is considerably lower than that of Vδ2 T cells, and healthy individuals' breast and lung tissues harbor a distinct subset of resident Vδ1 T cells [15, 16]. An investigation of immune cell repositories in healthy adults revealed that PD-1 expression in Vδ1 T cells is higher than that in Vδ2 T cells [17].

Generally, Vδ2 T cells amplified by zoledronic acid (Zol) and IL-2 have potential clinical efficacy as demonstrated by the ability to effectively eliminate cancer cells in vitro [17, 18]. Circulating adult Vγ9Vδ2 T cells express minimal levels of PD-1, which peaks at 3–4 days following activation and subsequently rapidly decreases to baseline levels within 7 days [19]. PD-1, TIM3 and CTLA-4 exhibit slight changes in expression within 14 days of amplification, but granzyme B (GZMB) is sharply upregulated on the third day and rapidly decreases [20]. During the amplification of Vδ2 T cells, the expression of ICPs increases in a Zol dose-dependent manner [21]. Similarly, the expression of inhibitory receptors during the amplification of Vγ2Vδ2 T cells exhibited upregulation on the third day, and PD-1 was expressed on the majority of Vγ2Vδ2 T cells. However, by the 14th day, Vγ2Vδ2 T cells lost the expression of PD-1 as well as CTLA4 [22].

Additionally, Vδ1 T cells amplified with IL-7 and phytohemagglutinin (PHA) demonstrated superior efficacy in killing cancer cells compared to Vδ2 T cells amplified by ZOL and IL-2. Furthermore, the expression levels of PD-1 and CTLA-4 are increased on Vδ2 T cells amplified with PHA and IL-7, while no such increase was observed in Vδ1 T cells [23].

### The function and immunosuppressive phenotype of γδ T cells in cancer patients

Numerous studies have documented a favorable association between the prognostic outcomes of patients with tumors and γδ tumor-infiltrating lymphocytes (TILs) [2]. However, the influence of immunosuppressive molecule expression on the functionality of these γδ TILs remains uncertain. In the context of cancer, γδ T cells undergo functional alterations, and the presence of PD-1 expressing γδ TILs has been observed in pancreatic ductal adenocarcinoma (PDAC), colorectal cancer (CRC), and multiple myeloma (MM) [24]. The expression of PD-1 serves as an indicator of γδ T cell functionality to a certain degree and can subsequently impact patient prognosis.

γδ T cells tend to accumulate at the T_CM_ stage in metastatic tumors of cancer patients. But following tumor progresses, there is a tendency toward an increase in the proportions of CD25^+^FoxP3^+^ regulatory γδ T cells in advanced disease compared to early stage. CD25^+^FoxP3^+^ regulatory γδ T cells are associated with poorer clinical outcomes. Additionally, the presence of LAG3, TIM3, PD-1 and 4-1BBL on peripheral γδ T cells and γδ TILs is related to early recurrence and shorter overall survival (OS) [25].

In an analysis of CRC samples, it was found that CD3 T cells accounted for an average of 20% of the total white blood cell (CD45) population. The percentage of B lymphocytes (6%) and NK lymphocytes (10%) is lower than that of CD3 T cells, while γδ T cells account for approximately 4.5% of the total white blood cell population [26]. In research on endometrial cancer and CRC, it was observed that γδ T cells express low levels of CTLA-4, PD-1, TIM3 and LAG3, akin to NK cells [27]. γδ T cells are more likely to constitute a subgroup of T cells with tumor cytotoxicity, and their immunosuppressive impact may be comparatively lower than that of other populations of antitumor cells (such as CD8, CD4 and mucosal-associated invariant (MAI) T cells) [28].

Additionally, γδ T cells are capable of sustaining elevated levels of cytotoxic effector molecules, such as GZMB, PRF1 and IFN-γ, similar to NK cells and CD8 T cells [27]. This observation aligns with previous findings from single-cell sequencing analysis of blood and tumor samples, which indicate that γδ T cells possess a mixed phenotype resembling both CD8 T cells and NK cells [28]. There are also studies demonstrating that γδ T cells infiltrating CRC exhibit more pronounced functional exhaustion than CD8 T cells or NK cells. Specifically, the levels of VISTA, PD-1 and TIM3 expressed by γδ T cells are much higher, while the levels of TNF-α and IFN-γ are considerably lower in γδ T than in NK cells or CD8 T cells in CRC [29]. Further studies have demonstrated that c-Maf promotes the upregulation of inhibitory receptors, thereby resulting in γδ T cell exhaustion [29].

Compared to those in healthy individuals, γδ T cells in tumor patients exhibit an increased immunosuppressive phenotype. In late-stage breast cancer samples, more than 20% of γδ TILs express CD73 and produce immunosuppressive cytokines (IL-8, IL-10, and adenosine) to facilitate tumor growth [30]. Furthermore, the ratio of peripheral γδ T cells in melanoma patients expressing TIM3, LAG3, 4-1BB, and ICOSL is higher than that in healthy donors, while the cytotoxic potential of γδ T cells is compromised both in PB and tumor tissue [25].

The proportion of PD-1^+^TIM3^+^ Vδ2 T cells is strongly higher in individuals with acute myeloid leukemia (AML) than in healthy controls [31]. Additionally, analysis of PB samples from AML patients in The Cancer Genome Atlas (TCGA) database revealed that elevated levels of FOXP3 and PD-1 co-expression on γδ T cells were associated with adverse clinical outcomes [32]. Similarly, γδ TILs in colon cancer mainly express PD-1^+^CD8α^−^ [33], in contrast to γδ T cells from PB and adjacent normal colon tissue, which display an effector phenotype but a diminished ability to secrete IFN-γ [26]. PD-1^+^CD8α^−^ γδ T cells preferentially express IL-17, while PD-1^−^CD8α^+^ γδ T cells preferentially produce IFN-γ [33]. The supernatant derived from cancer stem cells significantly inhibits the release of IFN-γ by γδ T cells while concurrently promoting the generation of IL-17 [26]. Furthermore, studies have indicated that Treg cells mediate the antitumor inhibition of γδ T cells in an IL-10 and TGF-β dependent manner [9]. More than 50% of CRC patients will experience colon liver metastatic (CLM). However, unlike in situ tumors, intrahepatic γδ TILs exhibit high cytotoxic potential in CLM, with low or negative expression of *PDCD1* and *CTLA4* [34].

The upregulation of PD-1 on γδ T cells can affect their potential toxicity to tumor cells. However, the mere increase in PD-1 expression alone cannot fully define the functional phenotype of γδ T cells in various tumor types. A comprehensive assessment involving other markers and indicators is necessary. In AML patients, PD-1^+^γδ T cells can produce more anti-tumor cytokines than γδ T cells lacking PD-1 or expressing both PD-1 and TIM3 [31].

γδ TILs in hepatocellular carcinoma (HCC) highly express the exhaustion marker gene *LAG3*, while *PD-1* and *TIM3* are not upregulated. Additionally, these cells express cytotoxic genes, including *IFNG, GZMB*, *NKG7* and *GNLY*, indicating that their status is an exhausted, yet cytotoxic population [35]. The proportions of peripheral γδ T cells in HCC patients and healthy individuals are comparable, but the number of γδ TILs is notably lower in HCC patients than in healthy liver tissue. This is evidenced by diminished G2/M cell cycle arrest and proliferation [35]. Compared to non-tissue-resident memory γδ T (γδ T_RM_) cells, γδ T_RM_ cells in liver cancer tissue exhibit higher levels of PD-1 expression. However, the majority of these cells demonstrate an enhanced capacity for rapid generation of IFN-γ and IL-2 upon stimulation [11]. Additionally, PD-1^high^ γδ T cells maintain their ability to secrete IFN-γ during stimulation. The expression of Eomesodermin (EOMEs) and Blimp-1 in γδ T_RM_ cells is relatively low, while the expression of Tcf-1, which endows T cells with a stem cell-like lifespan, trends to increase [11]. Hence, despite the increased expression of γδ T immunosuppressive molecules in the TME, certain cells can still maintain their heightened cytotoxic function. This observation aligns with the notion that CD8 T_RM_ cells expressing PD-1 do not represent a population that is functionally impaired [36].

### Immunosuppressive phenotypes of different γδ T cell subtypes in cancer patients

Different subtypes of γδ T cells can cause infiltration and functional changes in tumor patients. In healthy populations, the ratio of Vδ1/Vδ2 is usually less than 1. However, in many cancer patients, this proportion is the opposite [2]. We summarized the infiltration patterns of Vδ1 and Vδ2 T cells in different tumors in Table 1. Most research on the antitumor properties of Vδ2 T cells has focused primarily on the peripheral circulation. A greater abundance of peripheral Vδ2 T cells is significantly correlated with improved OS in patients. In triple negative breast cancer (TNBC) patients, Vγ9Vδ2 T cells in the PB exhibited increased PD-1, TIM3 and TIGIT expression. Additionally, when stimulated in vitro, these cells displayed diminished production of TNF-α and IFN-γ, as well as a slight reduction in the expression of GZMB and PRF1 [37]. The proportion of Vδ2 T cells in the PB of AML patients is significantly decreased. This decrease was accompanied by a decrease in NKG2D expression, an increase in PD-1 expression, and defects in IFN-γ generation. However, there was no significant decrease in the production of IL-2 or TNF-α, indicating that Vδ2 T cells were highly activated but exhausted during diagnosis [38]. Similarly, in melanoma patients, the abundance of circulating Vδ2 T cells also decreased. Notably, patients who died within 9 months had lower levels of Vδ2 T cells. Therefore, the baseline frequency of Vδ2 T cells holds promise as a candidate for predicting patient outcomes [39].

**Table 1:** The infiltration levels of γδ T cells and potential immunotherapy in cancer patients

Subsets	Cancer types	Locations	Infiltration level	Potential immunotherapies of cancer	References
Vδ1	Ovarian cancer	PB	Similar ratio with HDs	Combination therapy of multiple immune checkpoint inhibitors	[40]
		MA	Significantly higher than PB		
		Tumor	Significantly higher than PB		
	Acute myeloid leukemia	BM	No significant difference compared to HD	Combination therapy of multiple immune checkpoint inhibitors	[41]
	Myeloma	BM	Significantly higher than HDs		[41]
	Melanoma	PB	Higher frequencies than HDs	Ipilimumab	[39]
	Hepatocellular carcinoma	Tumor	Significantly higher than HDs	Allogeneic γδ T cell transfer in combination with LAG3 checkpoint blockade	[35, 46]
	Rectal cancer	Tumor	Significantly greater than in para-carcinoma tissues	Vδ2 T cell-based adoptive immunotherapy	[47]
Vδ2	Triple negative breast cancer	PB	Lower frequencies than HDs	Vγ9Vδ2 T cells and 1-MT (IDO1 inhibitor)	[48]
	Acute myeloid leukemia	PB	Decrease in proportion than HDs	PD-1 inhibitor, chimeric antigen receptor T-cell therapy, γδ T-based adoptive immunotherapies, bispecific antibody, anti-TRGV9/anti-CD123	[38, 41, 49]
		BM	Lower frequencies than HDs		
	Melanoma	PB	Lower frequencies than HDs	Ipilimumab	[39]
	Hepatocellular carcinoma	PB	Lower frequencies than HDs	Allogeneic γδ T cell transfer in combination with LAG3 checkpoint blockade	[35, 46]
		Tumor	Lower frequencies than HDs		
	Rectal cancer	Tumor	Significantly lower than in para-carcinoma tissue	Vδ2 T cell-based adoptive immunotherapy	[47]

*PB* peripheral blood, *HDs* healthy donors, *MA* malignant ascites, *BM* bone marrow

In comparison to Vδ2 T cells, Vδ1 T cells, which are characterized by solid tumor infiltration, diverse immunosuppressive molecules, and a predominantly T_EMRA_ phenotype, are more abundant in PT. Consequently, Vδ1 T cells potentially exert a significant influence on antitumor immunity. Notably, the Vδ1 T cell population is greater among γδ T cells within primary tumors and malignant ascites lymphocytes (MALs) in ovarian cancer (OC) [40], as well as resident in the bone marrow (BM) of AML and MM patients [41]. An increased percentage of Vδ1 T cells positive for FOXP3 [32], CD39, PD-1 and TIM3 was primarily observed, which is characterized by elevated co-expression of multiple immunosuppressive molecules with TIGIT [41]. The differentiation of Vδ1 T cells in TILs and MALs in OC differs: the majority of Vδ1 TILs exhibit a T_EM_ phenotype, while Vδ1 MALs exhibit a more mature T_EMRA_ phenotype. TIGIT and TIM3 are abundantly expressed in Vδ1 T cells in both MALs and PBLs, and PD-1, CD39 and OX40 have the highest expression in Vδ1 TILs [40]. Vδ1 T cells found in certain tumors exhibit elevated levels of immunosuppressive molecules but also express corresponding activation molecules.

A rare neuroendocrine skin cancer, Merkel cell carcinoma (MCC), is characterized by significant infiltration of Vδ2- T cells that express PD-1 and LAG3, as well as the activation and tissue retention markers CD69 and CD103 [42]. Similarly, Vδ1 T cells with effector memory and resident memory phenotypes are enriched in lung tumors, which are similar to stem cell-like CD8 T cells expressing EOMEs, TCF7, and TOX. The number of Vδ1 T_EMRA_ cells present in tumors and the number of CD103^+^ Vδ1 T cells residing in adjacent normal tissue are significantly correlated with sustained remission in patients after surgery [15]. Additionally, these Vδ1 T cells are related to the non-progression and OS of patients with TNBC [16]. In the peritumoral compartment of CLM patients, Vδ1 T cells also constitute the predominant population of TILs and are characterized by high expression of CD69, which is indicative of effector cell function. These Vδ1^+^CD69^+^ TILs are associated with improved clinical outcomes in patients [34].

The γδ T cell subtypes within a given tumor exhibit distinct functional variations, characterized by heightened plasticity and functional diversity. Notably, liver cancer patients exhibit a significant reduction in the percentage of tumor-infiltrating and circulating Vδ2 T cells and a significant increase in the percentage of Vδ1 T cells [35]. The Vδ1 T subgroup exhibited a substantial decrease in NKG2D expression, while PD-1 expression remained unaltered. Conversely, Vδ2 T cells display a marked increase in PD-1 expression, consequently impairing antitumor immune responses [43].

Among renal cell carcinoma (RCC) patients, Vδ2 T cells are the main group expressing PRF1 and GZMB, while Vδ2^−^ T cells (include Vδ1 and Vδ3 T cells) are more abundant in tumors and almost absent in healthy tissues. These Vδ2^−^ T cells exhibit a unique phenotype characterized by early activation markers (CD28 and CD27) and effector cell-related markers (PD-1, CD57, and 4-1BB). Vδ2^−^ T cells are also the primary group of γδ T cells expressing PD-1, TIM3, and TIGIT, but they maintain the expression of effector cytokines at levels similar to those of unexhausted cells and are capable of killing autologous tumor cells in vitro [24]. These findings are not entirely consistent with the above findings that Vδ1 T cells strongly infiltrate the tumors of CRC patients [44]. Compared with Vδ2 T cells, PD-1 expression in Vδ1 T cells is lower, but TIGIT and secreted GZMB, GZMK, and TNF levels are higher [27], indicating strong cytotoxic gene expression. However, Vδ1 T cells in tumors exhibit genetic features related to metabolic adaptation and tumor-promoting functions [33], which are correlated with patient stage and prognosis [44].

Specifically, DNA mismatch repair-deficient (dMMR) cancer contains β2M mutations that prevent β2M recognition by T cells, increasing the likelihood of immune escape. Recent studies have revealed a strong association between β2M inactivation in dMMR colon cancer and Vδ1 and Vδ3 T subpopulations that have high expression of PD-1 and other activation markers, as well as higher expression of killer cell immunoglobulin-like receptors (KIRs) compared with Vδ2 T cells. These PD-1^+^γδ T cells exhibit increased sensitivity to β2M knockout tumor-like organs and human leukocyte antigen (HLA) class I-negative dMMR colon cancer cell lines compared with cells with normal antigen-presenting function [45].

In CLM, high transcriptional levels of inhibitory KIRs exist on Vδ1 and Vδ3 TILs, while NKG2A and KLRG1 are expressed on Vδ2 TILs. Unlike in the original cancer, TIGIT is highly expressed on Vδ3 T cells [34]. Therefore, different types of cancer and different sites may have different γδ T cell subtypes, which may result in different phenotypes, functional changes, and clinical outcomes.

## The correlation between the PD-1/PD-L1 axis and the immunosuppression of γδ T cells

The PD1/PD-L1 axis is a crucial pathway for the immune response in various types of cancer. By inhibiting phosphatase containing SHP2 to the immunoreceptor tyrosine-based switch motif (ITSM) at the tail of PD-1, PD-1 hampers the function of T cells. Positive signals triggered by TCR (interacting with MHC-I peptides) and CD28 (interacting with CD86 and/or CD80) can be reversed by these phosphatases. Furthermore, PD-1 increases the expression of the basic leucine zipper transcription factor ATF-like (BATF), which inhibits T cell function [50]. As mentioned earlier, inhibitory receptors expressed on γδ TILs [51, 52] and amplified Vδ2 T cells inhibit T cell antitumor function when interacting with the ligands of these inhibitory receptors [22].

**Fig. 1:**
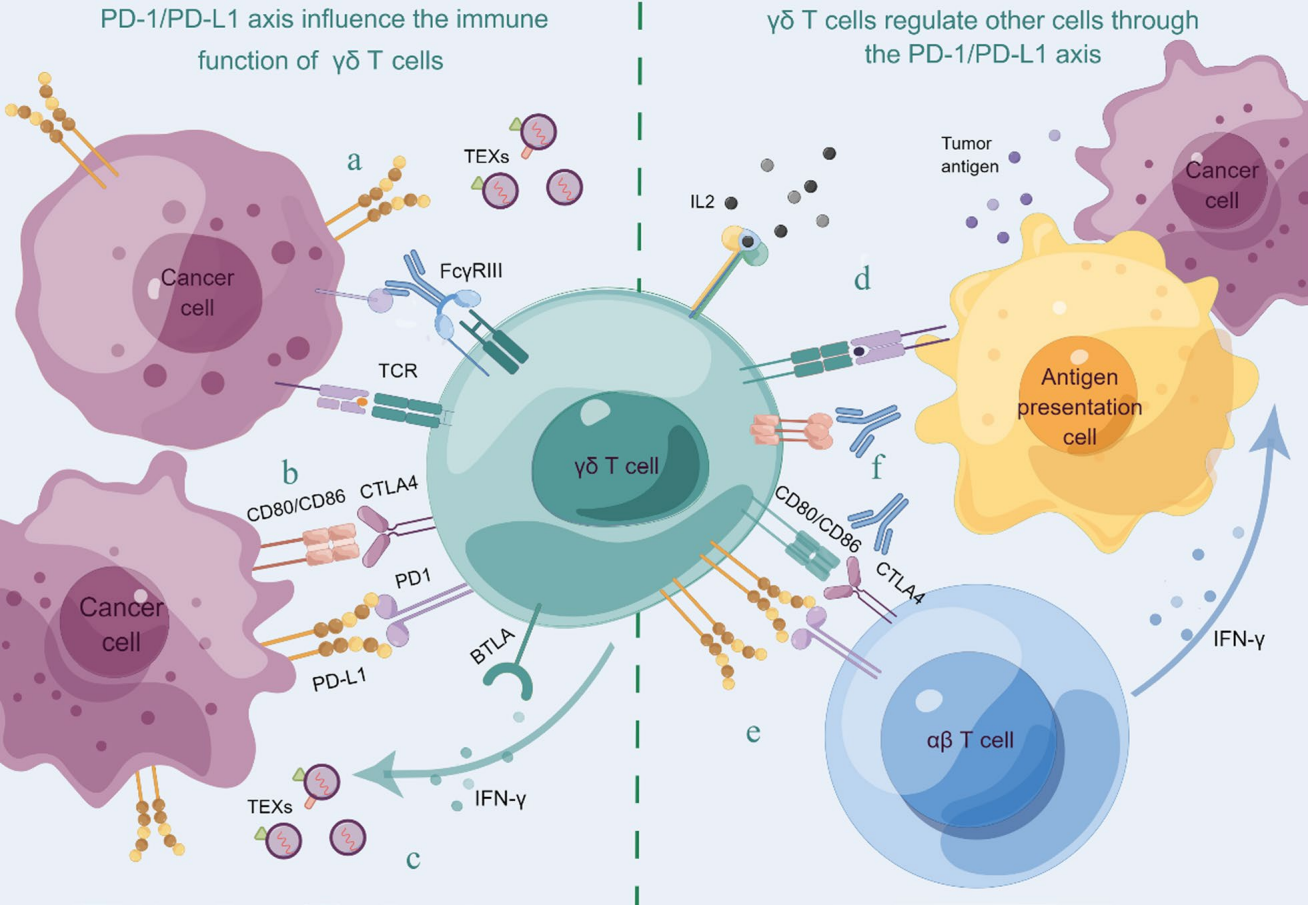
The PD-1/PD-L1 axis influences the immune activity of γδ T cells: **a**. The PD-1/PD-L1 axis weakens the TCR-mediated activation of γδ T cells and the ADCC effect; **b**. PD-L1 and CD80/CD86 weakens the antitumor function of γδ T cells by binding to immune checkpoint molecules (PD-1, CTLA4, BTLA) on the surface of γδ T cells; **c** TEXs promote the activation of γδ T cells, and the IFN-γ secreted by activated γδ T cells promotes the upregulation of PD-L1 in tumor cells, enhancing the inhibitory effect on γδ T cells. Immune regulation of γδ T cells on other cells through the PD-1/PD-L1 axis: **d** IL-2 and APC can enhance the immunosuppressive potential of γδ T cells; **e** the interaction between PD-L1 or CD80/CD86 expressed on γδ T cells and αβT cells weaken the antitumor effect of αβ T cells; **f** blocking CTLA4 and TLR2 can partially eliminate the inhibitory effect of γδ T cells

Research has clarified that even without the use of ICIs, Vδ2 T cells amplified from human PBMCs still have effective antitumor effects after adoptive transfer [19]. Simultaneously, Vδ2 T cells after cryopreservation still retain most of their antitumor function in vivo, indicating that they can be employed in preclinical studies and clinical treatments [22]. However, Vδ2 T cells are different from CD8 T cells. Despite the fact that the number of Vδ2 T cells had increased 62 folds, their antitumor immunity was not improved with the adoptive transfer of more Vδ2 T cells [22]. This observation indicates that the TME may act a key part in influencing γδ T cells to control tumor growth (Fig. 1): some TMEs are in an immunosuppressive state, which affects the immunotherapeutic effect of γδ T cells; IFN-γ or other cytokines released by Vδ2 T cells may enhance PD-L1 expression on tumor cells during tumor recognition, potentially reducing antitumor immunity [22]; Tumor cells are not the only producers of isopentenyl pyrophosphate (IPP), which can activate γδ T cells through TCR in the TME, and BM-derived stromal cells (BMSCs) in monoclonal gammopathy of undetermined significance (MGUS) and MM also release a significant amount of IPP. Therefore, chronic TCR conjugation in the immunosuppressive TME (characterized by inappropriate cytokines and/or co-stimulatory signals) may lead to PD-1 expression and γδ T cell dysfunction [10]; γδ T cells can also induce an immunosuppressive state in other antitumor cells through PD-L1.

### PD-1/PD-L1 axis influence the immune activity of γδ T cells

Compared to that in healthy individuals, the number of PD-L1^+^ myeloid suppressor cells (MDSCs) is increased in MM patients, while Vγ9Vδ2 T cells are surrounded by PD-L1^+^ myeloma cells. TNBC patients also exhibit high PD-L1 expression, which leads to exhaustion of Vγ9Vδ2 T cells [37]. The BM microenvironment significantly hinders the phosphoantigens (pAgs) reactivity of these T cells, further inducing γδ T cells exhaustion [53], which downregulates their cytotoxicity, IFN-γ generation, and ADCC through the PD-1/PD-L1 axis [10]. Dysfunction of Vγ9Vδ2 T cells is an early and persistent event that can be identified in patients with MGUS and these patients may not fully recover during the clinical remission period after autologous stem cell transplantation [53, 54]. Anti-TGF-β or OX-40L can be utilized to inhibit intratumoral regulatory T cells (Tregs) in the TME but these treatments are unable to restore the proliferation of Vγ9Vδ2 T cells in the BM [53]. PD-1 expression on Vγ9Vδ2 T cells in the BM of myeloma patients increased after Zol stimulation. That is, PD-1 is upregulated to increase resistance to pAg-induced TCR activation [10]. However, when primary AML cells sensitized by Zol were incubated with γδ T cells, it was demonstrated that tumor cells can induce γδ T cells to express PD-1. However, these PD-1^+^ γδ T cells exhibit increased levels of IFN-γ, which proves that they still have certain functions [52]. It has further been reported that PD-L1 indirectly regulates γδ T cells. The oxygen pressure in the TME can alter the content of tumor derived exosomes (TEXs). Hypoxia can inhibit TEX-stimulated γδ T cell activity, further coordinating the balance between pro- and antitumor γδ T cells, and promoting MDSC mediated inhibition of γδ T cells via the miR-21/PTEN/PD-L1 axis [55].

Notably, B lymphocyte and T lymphocyte attenuators (BTLA) exhibit robust expression on γδ T cells in leukemia patients. BTLA, a member of the CD28 family, interacts with its ligand herpesvirus entry mediator (HVEM). Like PD-1, BTLA negatively regulates T cell activation. Despite both BTLA and PD-1 possessing ITSM and ITIM motifs, PD-1 predominantly relies on ITSM to achieve its inhibitory function, whereas BTLA necessitates the concurrent presence of both ITIM and ITSM [56]. Resting γδ T cells, especially naïve γδ T cells, express high levels of BTLA, which is downregulated in the T_CM_ and T_EM_ stages. In contrast, PD-1 is upregulated in γδ T_EM_ cell subpopulations. Additionally, PD-1 expression increases after TCR involvement, while BTLA expression is markedly attenuated. Exposure to lymphoma cells leads to a notable decrease in γδ T cell growth through BTLA/HVEM interactions, and inhibiting BTLA/HVEM signaling can enhance γδ T cells proliferation [57]. However, manipulating BTLA/HVEM signal transduction does not change the effect of γδ T cells on target cell death during cultivation or the reaction period. These findings demonstrate the distinct regulation of BTLA and PD-1 expression, potentially indicating divergent functions [57]. BTLA governs the proliferation of γδ T cells, while PD-1 regulates their cytotoxicity [58].

### Immune regulation of γδ T cells on other cells through the PD-1/PD-L1 axis

The expression of PD-L1 on γδ T cells in patients with tumors has implications for the functionality of other cells within the TME. Specifically, the expression of PD-L1 in γδ TILs of oral cancer patients surpasses that in PTs, while there is a significant increase in PD-L1 expression on γδ T cells in the PB of these patients compared with healthy controls [59]. Moreover, in patients with PDAC, PD-L1 and galactose lectin-9 are increased in circulating γδ T cells and γδ TILs, resulting in the promotion of tumor growth by inhibiting the cytotoxic activity of CD4 and CD8 T cells [60]. The co-culture of CD8 T cells and γδ T cells isolated from the PB of healthy donors markedly increase the proportion of CD8 T cells undergoing apoptosis when subjected to IL-2 stimulation and hypoxia [59]. Consequently, IL-2 and hypoxia have the potential to augment the PD-L1 expression on γδ T cells, affecting the survival of CD8 T. Furthermore, stimulation with IL-12/18 can also result in an increased expression of co-inhibitory receptors and PD-L1 on γδ T cells, along with heightened phosphorylation of JNK and p38 in γδ T cells. The upregulation of co-inhibitory receptors remains largely unaltered following the administration of p-JNK or p-p38 inhibitors. However, the use of a p-JNK inhibitor (SP600125) considerably reduced the PD-L1 expression on γδ T cells, without concomitantly increasing of IFN-γ and GZMB expression on CD8 T cells. Consequently, the observed decrease in PD-L1 expression on human γδ T cells is more likely to contribute to a reduction in the immune suppression of CD8 T cells in vivo [61].

In conclusion, PD-L1 expression on γδ T cells has the potential to act as a regulatory mechanism for T cells who exhibit a response. The inhibitory impact of PD-L1 can be counteracted to a certain extent by anti-PD-L1 antibodies, while anti-PD-1 antibodies have relatively limited effects. It is plausible that, in addition to the inhibitory PD-1, there may be unidentified PD-L1 secondary co-stimulatory receptors [62].

In addition to PD-L1, Vδ2 T cells exhibit immunosuppressive capabilities when exposed to antigen-presenting cells or when co-stimulated with an anti-CD28 monoclonal antibody (mAb). This immunosuppressive potential can be counteracted by Toll-like receptor 2 (TLR2) ligands or a substantial quantity of Th1 cytokines produced by reactive T cells [62]. Consequently, the inhibition effect of αβ T cells can be eliminated by obstructing the interaction between CD86 of Vδ2 T cells and CTLA-4 of αβ T cells. Using TLR2 ligands pre-treatment Vδ2 T cells has been shown to augment the phosphorylation of NF-κB, MAPK and AKT, thereby partially attenuating their inhibitory effects. Additionally, co-cultured responder T cells exhibit downregulation of inhibitory molecules, accompanied by restoration of the phosphorylation of AKT and NF-κB [62]. Moreover, anti-CD80 therapy does not affect the interaction between Vδ2 T cells and responsive T cells [62]. The modulation of αβ T cells by activated Vδ2 T cells has the potential to refine αβ T cells responses, thus offering potential utility for the regulation of responsive T cells in cancer patients through the manipulation of γδ T cells or elimination of inhibitory γδ T cells from cells used for adoptive transfer.

## The effect of regulating the PD-1/PD-L1 axis on γδ T cells phenotype and function

### The effect of immune checkpoint inhibitors (ICIs) on γδ T cells function

The US-FDA approved ICB therapy for the treatment of a variety of cancer types. PD-1/PD-L1 blockers have emerged as the primary medication option for various tumors, either as a standalone treatment or in combination with CTLA-4 blockers, chemotherapy, or targeted therapy [63]. Although blocking antibodies targeting PD-1 or PD-L1 have demonstrated considerable success in clinical trials, a majority of patients do not exhibit enduring remission following PD-1 treatment [64]. As an important subgroup of antitumor therapy, γδ T cells require further understanding of the impact of ICBs on their function.

#### The effect of an anti-PD-L1 antibody on γδ T cells

Anti-PD-L1 mAb treatment can augment the antitumor effect of γδ T cells on PD-L1^+^ Daudi cells [65], as well as on Zol-pretreated bladder cancer cells (UMUC3, TCCSUP, T24 and T24cis), breast cancer cells (MDA-MB-231) and mesothelioma cells (H2052 and H2452) with high expression of PD-L1 [66]. However, the use of anti-PD-L1 mAb has been shown to augment the ADCC activity of γδ T cells to target cancer cells characterized by high PD-L1 levels [67]. To exclude the influence of ADCC, PD-L1 in tumor cells was silenced, which did not increase the cytolytic activity of γδ T cells [66]. Studies in liver cancer have shown that the co-administration of anti-PD-L1 blockers and γδ TCR stimulation in vitro does not substantially elevate the generation of IFN-γ or GZMB in Vγ9Vδ2 TILs [11]. Moreover, the utilization of anti-PD-L1 mAb in TNBC patients does not further enhance the antitumor immunity of Vγ9Vδ2 T cells [37].

Additionally, expanding Vδ2^−^T cells in RCC have been found to exhibit comparable levels and kinetics of autologous tumor cell killing to those of CD8 T cells but the antitumor activity of anti-PD-L1 mAbs is not significantly enhanced in vitro [24]. Nevertheless, research suggests that the combination of anti-PD-L1 mAb and the adoptive transfer of IL-12/18/21 pre-activated γδ T cells can enhance their cytotoxicity function in a CD8 T cell dependent manner. This combination promotes the secretion of TNF-β and IFN-γ by CD8 T cells [68]. Patients with locally advanced and metastatic urothelial carcinoma were treated with atezolizumab in a clinical trial (NCT02108652) to assess its efficacy, and to analyze 168 tumor samples. The analysis demonstrated a significant correlation between Vδ2 genetic characteristics and favorable clinical response [24]. Therefore, the effect of anti-PD-L1 mAbs on γδ T cells needs to be considered with the corresponding effects on the tumor environment. When PD-L1 is highly expressed in the TME, administration of anti-PD-L1 monoclonal antibodies will likely augment their antitumor efficacy.

#### The effect of anti-PD-1 antibody on γδ T cells

In tumors with immunosuppressive environments, anti-PD-1 therapy can promote the killing function of γδ T cells against tumors. Stimulation of Vγ2Vδ2 T cells with pamidronate to treat prostate cancer increases PD-L1 expression on PC-3 cells. Consequently, the concurrent utilization of PD-1 checkpoint inhibitors and the adoptive transfer of Vγ2Vδ2 T cells have significantly enhanced antitumor immune responses, resulting in a substantial reduction in tumor volume, approaching complete eradication [22]. However, some studies have shown that the inhibition of PD-1 in bladder cancer cells, breast cancer cells and mesothelioma cells with high PD-L1 expression does not enhance the killing function of γδ T cells [66].

In research on hematological tumors, although it was observed that blocking PD-1 did not significantly affect the direct cell-mediated destruction of leukemia cells by γδ T cells, it did significantly augment in the synthesis of IFN-γ within the cytoplasm of γδ T cells [52]. With the increase in the anti-PD-1 mAb concentration in MM, Vγ9Vδ2 T cells stimulated with Zol in the BM partially resumed proliferation, with a nearly fivefold increase in cytotoxic potential and upregulation of CD107a expression [53].

Therefore, although PD-1 checkpoint inhibitors did not enhance cytolysis by stimulated or expanded γδ T cells, they effectively induced the secretion of IFN-γ and pro-inflammatory cytokines. Other studies that were conducted demonstrated that inhibiting PD-1 alone does not have a substantial impact on the generation of TNF-α and IFN-γ in Vδ2 T cells. However, the inhibition of TIM3 and the dual inhibition of PD-1/TIM3 have been demonstrated to significantly enhance their production.

This phenomenon may be attributed to the compensatory increase in TIM3 expression following the use of anti-PD-1 antibodies [31]. It should also be acknowledged that the use of a single PD-1 blockade may exacerbate the regulation/inhibitory polarization of Vγ9Vδ2 T cells in MM by inducing the release of inhibitory factors such as IL-10 and the expression of other inhibitory molecules (CD73, FOXP3, CD38 and CD39) [10]. Specifically, some patients with follicular lymphoma (FL) exhibit a “high” immune escape phenotype, with extensive infiltration of PD1^+^CD16^+^ Vγ9Vδ2 T lymphocytes. Unlike PD-1, which is induced by high levels of PD-L1 expression in solid tumors, most γδ TILs with ADCC express PD-1, which influences the cytolytic activity of γδ T cells toward FL cell aggregates. The inhibition of PD-1 enhances the innate cytotoxicity of FL and ADCC [69].

In clinical practice, the therapeutic effect of PD-1 antibodies on γδ T cells is positive. By comparing paired tumor samples obtained from dMMR colon cancer patients before and after dual blockade of PD-1 and CTLA-4, it was shown that ICB significantly increased the number of γδ TILs, indicating that γδ T cells are crucial for the ICB response in HLA class I negative dMMR colon cancer patients [45, 70]. At the mid to late stages of treatment with ipilimumab alone in melanoma patients, the proportion of Vδ2 T cells remains unchanged or increases, which is related to better OS and higher clinical benefits for patients [39]. Four MCC patients who received ICI treatment had a high tumor γδ T cell enrichment score, three of which responded completely to the treatment. After resection of the retroperitoneal tumor mass, the patient received adjuvant treatment and remained disease-free after 3 years of continuous treatment [42]. RNA-seq data analysis of a phase II basket clinical trial (NCT02644369) of pembrolizumab in advanced solid non-small cell lung cancer (NSCLC) revealed that patients with *TRDV1* expression higher than the median in tumor biopsy before treatment had a significant increase in survival rate, indirectly reflecting that high Vδ1 T cells infiltration is beneficial for patient prognosis [15]. It should also be noted that there have been reports of cases during long-term Hodgkin lymphoma (HL)-associated inflammation, where blocking PD-1 accelerates γδ T cells clone proliferation and leads to secondary hyperplastic T cells lymphoma [71]. These studies further indicate that the combined blockade of PD-1 and other immune checkpoints on γδ T cells can enhance their antitumor cytotoxicity potential, which seems to be an effective strategy for enhancing immunity.

### The effects of cytokines and drugs on immune checkpoint molecules and γδ T cells functions

The prevailing notion suggests that Zol-induced TCR triggering can partially counteract the inhibitory impact of PD-1 on γδ T cells. Upon exposure to Zol-treated PD-L1^+^ tumor cells, PD-1^+^ γδ T cells undergo of γδ TCR-mediated signaling. This induction leads to a mild to moderate reduction in cytokine production compared to that induced by PD-L1^−^ tumor cells. Nevertheless, PD-1^+^ γδ T cells exhibit similar cytotoxic activity against both PD-L1^+^ and PD-L1^−^ tumor cells treated with Zol [65]. Stimulation of Vδ2 T cells with IL-2 and the phosphate antigen 4-hydroxy-3-methyl-but-2-enyl-pyrophosphate (HMBPP) for 24 h resulted in a significant upregulation of the PD-1^+^TIM3^+^ subgroup. This subgroup exhibited the lowest production levels of TNF-α and IFN-γ [31]. Additionally, the use of TCR pan-γδ antibodies can also amplify γδ T cells. PD-1 expression is rapidly induced upon activation for 48–72 h. However, the expression of PD-1 gradually decreases and stabilizes to the initial level after transferring cells to wells without the presence of an antibody [72].

In conjunction with the utilization of IL-2 and Zol for the co-amplification of Vγ9Vδ2 T cells intended for adoptive transfer, the inclusion of the corresponding cytokines can produce twice the result with half the effort. The utilization of cytokine combination pre-treatment strategies, such as IL12/18, IL12/18/21, IL12/15/18, or IL12/15/18/21, has been clarified to dramatically augment the cytotoxicity and activation of γδ T cells in vitro. Additionally, these methods have been shown to modulate the function of γδ T cells and regulate the expression of immunosuppressive molecules on γδ T cells [68].

The administration of IL-12/18 resulted in elevated PD-1, CTLA-4, TIM3 and LAG3 expression in Vδ2 T cells, along with significant upregulation of activation related genes (*IFNG*, *FASLG*, and *GZMB*). These compounds increase the expression of IRF4, BATF and signallng lymphocytic activation molecule (SLAM) family member 7, which can induce a high level of IFN-γ and increase the killing of tumor cells [61]. Moreover, EOMEs^+^ γδ T cells exhibit increased proliferation rates and increased IFN-γ generation efficiency. However, γδ T cells with excessively high expression of EOMEs exhibit a exhaustion phenotype of PD-1 [73]. Furthermore, the combination of IL-12/IL-4 in γδ T cells has been shown to elicit the expression of EOMEs. The use of Zol, IL-2, IL-15, and vitamin C for Vγ9Vδ2 T cells expansion in vitro can better promote their proliferation and differentiation. In comparison to expansion solely with Zol and IL-2, this approach leads to significantly elevated expression of co-stimulatory molecules, heightened levels of effector molecules (NKG2D, TNF-α and IFN-γ) and CD107a, and enhanced cellular energy metabolism. Moreover, they have improved antitumor function in vitro [18]. Researchers have also discovered that amplified Vδ1 T cells, derived from healthy blood, produce amphiregulin (AREG), which possesses wound healing capabilities. However, researchers have found that a combination of IL-15, IL-18, anti-CD3, and anti-CD2 can effectively sustain the expansion of antitumor cytotoxic Vδ1 T cells, while concurrently limiting the amplification of a specific subset that produces AREG, thereby promoting tumor wound healing. This approach ultimately leads to improved tumor infiltration and clearance [27].

Compared with those in the Zol treatment group, the percentages of total and PD-1^+^ Vδ2 T cells generated by live Bacille Calmette-Guérin (BCG) stimulation markedly decreased [74]. BCG may lead to weaker stimulation, inducing lower activation levels and resulting in the downregulation of PD-1. Alternatively, the multiple fold increase in IFN-γ induced by BCG stimulation compared to that induced by Zol stimulation may lead to a substantial increase in PD-L1 expression. Consequently, PD-1 binds to PD-L1, facilitating the negative selection of PD-1^+^ cells [13]. Moreover, BCG amplified cells exhibit a greater degree of degranulation upon recognition of tumor cells, resulting in the production of a wider range of cytokines [21]. Considering that BCG is an effective inducer of the Th1 response to control *Mycobacterium*-mediated bladder tumors, the concurrent administration of Zol and BCG has the potential to enhance the proportion and multifunctionality of bladder tumor reactive Vδ2 T cells. This combination therapy may be an effective treatment option for non-muscle-invasive bladder cancer patients [75]. 1α, 25(OH)_2_D_3_ can promote the nuclear translocation of vitamin D receptor (VDR), which can bind to the promoter regions of the *TIGHT, PDCD1* and *TIM3* genes to inhibit their expression. Moreover, it can also upregulate CD28. Pretreatment with 1α, 25(OH)_2_D_3_ increases Th1 cytokine production in Vγ9Vδ2 T cells and enhances antitumor immunity [76].

The prolonged utilization of indomethacin has been observed to induce the upregulation of PD-L2 and PD-1 in γδ T cells, in a dose-dependent manner, via the TRIF/NF-κB and JAK/STAT3 pathway, while concurrently suppressing the generation of effector molecules in hepatocellular carcinoma [77]. Similarly, histone deacetylases (HDACs) can modify the acetylation of histones within chromatin, thereby augmenting gene transcription. HDAC inhibitors, which are employed as therapeutic agents against tumors, have been found to impede tumor progression, promote cellular apoptosis, and facilitate cellular differentiation [78]. The tumor cells they treat are easily killed by γδ T cells, but it suppresses γδ T cells antigen-specific proliferation and antitumor effects are suppressed by the upregulation of immune checkpoints (PD-L1 and PD-1). Reductions in the expression of effector molecules, activation markers CD69 and CD25, and transcription factors (T-bet and EOMEs) expression are also observed on γδ T cells.

## Combination therapy targeting ICPs to enhance γδ T cells antitumor function

### Ameliorating the immunosuppression of γδ TILs

**Fig. 2:**
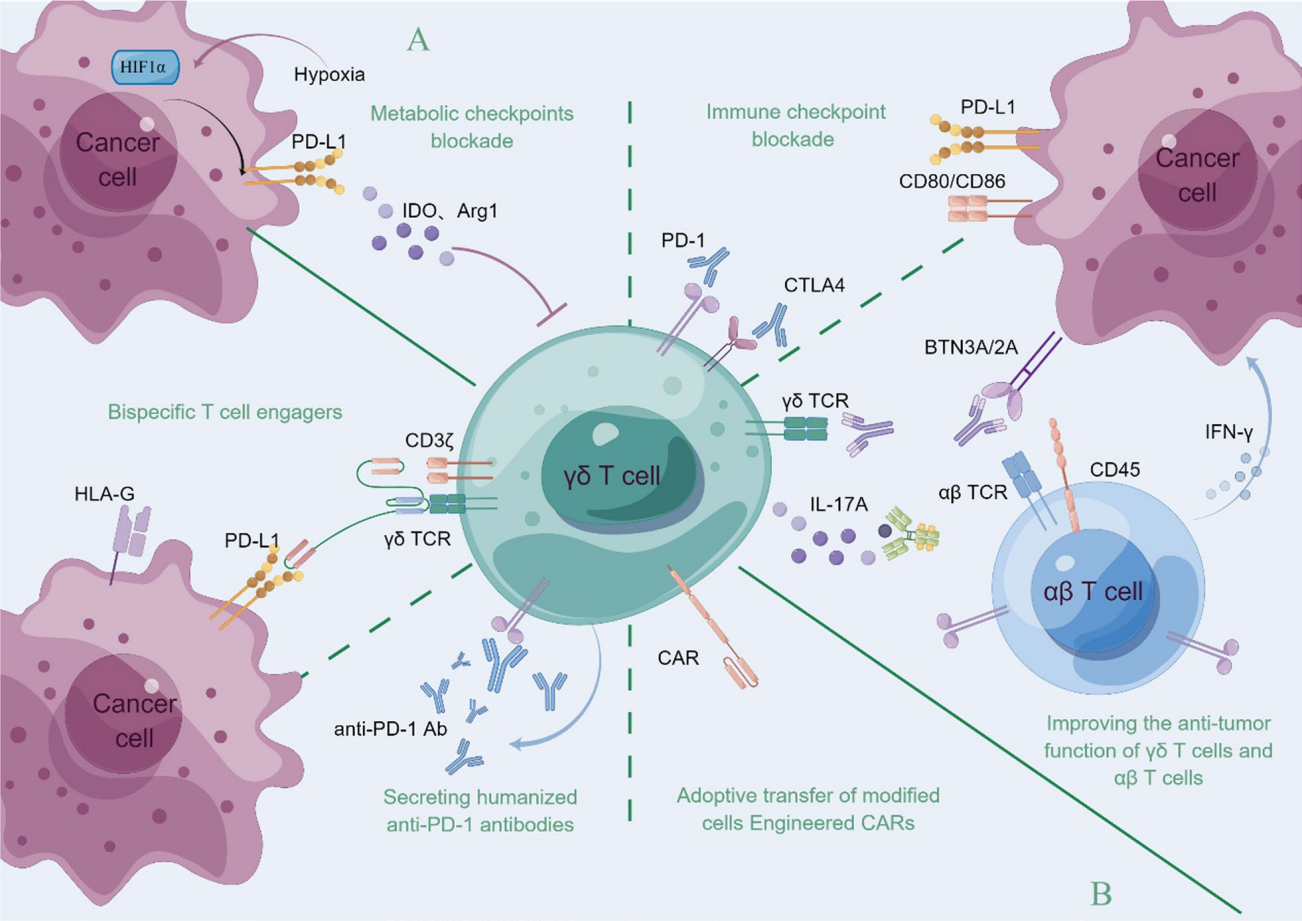
**A** Ameliorating the immunosuppression of γδ TILs: combining ICPs with metabolic checkpoint inhibitors, and mAbs (IL-17A mAb, BTN3A mAb, etc.). **B** Enhancing the therapeutic effect of γδ T cells adoptive transfer: adoptive infusion of CAR γδ T cells, construction of γδ T cells secreting humanized anti-PD-1 antibodies, and construction of a bispecific T-cell engager (bsTCE) to activate γδ T cells

Targeted modulation of ICPs can ameliorate the impaired functionality of exhausted γδ T cells within the TME, thereby exerting antitumor effects through enhanced proliferation, activation and cytotoxicity [79]. The prospect of combining ICIs with chemotherapy drugs, mAbs, small molecule inhibitors, and other therapeutic agents holds promise for ameliorating γδ T cells immunosuppression (Fig. 2).

To mount effective immune responses in the face of specific challenges, γδ T cells must undergo metabolic resetting. This involves the significant downregulation of functional pathways associated with γδ TILs, including OXPHOS, glycolysis, and fat and amino acid metabolism pathways. Conversely, glutamine metabolism and its related pathways (nitrogen, arginine, and proline metabolism) are upregulated [35]. By targeting specific metabolic checkpoints (MCPs) and combining them with ICP/ICP-L blockade, it is possible to enhance γδ T cells function and improve the efficacy of immune intervention in cancer treatment. The expression of glucose transporters on the surface of γδ T cells is higher than that on the surface of αβ T cells, suggesting a greater reliance on glucose uptake and metabolism. Combining ICP/ICP-L inhibitors with lower therapeutic concentrations of glycolytic inhibitors, to mitigate side effects, can synergistically enhance the antitumor activity of γδ TILs. ICP/ICP-L inhibitors can also be used in conjunction with AKT/mTOR inhibitors to facilitate a more balanced redistribution of glucose in the TME [80].

Although γδ T cells can survive in hypoxic microenvironments, their ability to exert antitumor cytotoxicity is compromised, resulting in the differentiation of γδ T17 cells. The exhaustion phenotype of PD-L1^high^γδ T cells in tumors is mediated by the hypoxic environment, suggesting that combination therapy targeting HIF1-α and blocking PD-1/PD-L1 signaling may effectively reverse hypoxia-induced immunosuppression [59]. Additionally, arginine deprivation has been found to inhibit the Vγ9Vδ2 T cell-mediated antitumor immune response thus the inhibition of arginase 1 (Arg1) can restore the capability of γδ T cells to secrete IFN-γ and effectively eliminate MDSC-induced immunosuppressive Daudi and Jurkat cells [80]. An IDO inhibitor (1-MT) can also rescue dysfunctional or exhausted T cells by increasing tryptophan levels and enhancing the cytotoxicity of Vγ9Vδ2 T cells by increasing the generation of PRF1 in TNBC [37].

The simultaneous enhancement of the antitumor capabilities of αβ T cells and γδ T cells through combination therapy exhibits considerable clinical applicability. The administration of vascular endothelial growth factor receptor tyrosine kinase inhibitor (VEGFR2-TKI) at high doses has been found to induce the generation of IL-17A in γδ T cells via the VEGFR1/PI3K/AKT pathway. This, in turn, promotes the differentiation of neutrophils into the N2 phenotype and accelerates the dysfunction of CD8 T cells, resulting in an increase in PD-1 expression [81]. However, the combination of VEGFR2-TKI with immunotherapy has been shown to alleviate tumor resistance caused by high-dose VEGFR2-TKI therapy. Additionally, the combination of an anti-IL-17A mAb and an anti-Ly6G mAb has been found to reduce the indirect inhibitory effect of γδ T cells on CD8 T cells and effectively decrease PD-1 expression [81, 82]. The molecular attributes of Vδ2^−^ demonstrate a significant correlation with the clinical response observed in patients with metastatic RCC who undergo combination therapy involving atezolizumab (anti-PD-L1) and bevacizumab (anti-VEGF) [24]. In NSCLC patients, the estrogen receptor α can predict the response to pembrolizumab and is positively associated with PD-L1 expression. The use of an aromatase inhibitor (letrozole) significantly improved the efficacy of pembrolizumab in immune-PDXs, increasing the frequency of antitumor Vγ9Vδ2 T and CD8 T cells. Therefore, aromatase can be used as an adjuvant for immunotherapy [83]. In addition, butylphilic protein (BTN) molecules are members of the B7 immunoglobulin superfamily and play an important role in stimulating γδ T cells [84]. In patients who exhibited non-responsiveness to anti-PD-1 treatment with pembrolizumab or nivolumab, the levels of circulating PD-1 and BTN2A1 prior to treatment significantly increased and decreased, respectively [85]. There is a discernible correlation between BTN2A1 and immune evasion. By combining BTN3A1 and BTN2A1 with TCRs, γδ T cells can be activated and their corresponding phenotypic characteristics can be altered, enhancing their antitumor effects [86]. BTN2A/3A binding antibody-clone 20.1, and the excitatory anti-BTN3A1 antibody-CTX-2026 [87] can activate most Vγ9Vδ2 T cells without exogenous pAg. Currently, a humanized anti-BTN3A agonist known as ICT01 is being evaluated in an interventional, nonrandomized phase I/II clinical study for the treatment of recurrent/refractory patients diagnosed with advanced or recurrent cancer by histology or cytology [88]. By employing anti-BTN3A1 to target Vγ9Vδ2 T cells and integrating it with ICP therapies, it becomes feasible to not only strengthen the antitumor functionality of Vγ9Vδ2 T cells, but also hinder the immunosuppressive impact resulting from the interaction between BTN3A1 and CD45-N-mannosylated residues on human αβ T cells [87]. Additionally, by obstructing the PD-1/PD-L1 axis, exhausted αβ T and Vγ9Vδ2 T cells can be revived, thereby enhancing their adaptive immune capacity [89].

### Enhancing the therapeutic effect of γδ T cells adoptive transfer

Currently, adoptive transfer therapy involving γδ T cells has achieved a degree of success in terms of clinical safety and therapeutic efficacy [18]. Nonetheless, there are several obstacles that need to be addressed, including the restricted accessibility of γδ T cells and their rapid exhaustion following repeated activation in vitro, inadequate comprehension of γδ TCR diversity and interactions with receptor ligands, and an underestimation of the influence on functional diversity [90]. Consequently, innovative treatment approaches could involve combining γδ T cells adoptive transfer therapy with ICIs [91], as well as combining it with antitumor medications and other immunomodulatory antibodies [54].

To enhance local immune suppression within tumors, researchers constructed γδ T cells capable of producing humanized anti-PD-1 antibodies, denoted as “Lv-PD-1-γδ T” cells. This novel approach demonstrates superior efficacy in mitigating local immune suppression compared to the combination of PD-1 antibodies and γδ T cells. In murine models of ovarian tumors, Lv-PD-1-γδ T cells exhibit enhanced cytotoxicity and enhanced proliferation, resulting in notable therapeutic outcomes and improved survival rates [72].

γδ T cells can effectively target tumor cells and augment their cytotoxicity. To this end, scientists have devised a Y-body-based bispecific antibody (bsAb) known as the Vγ2 × PD-L1 antibody, which exhibits a selective affinity towards Vγ2Vδ2 T cells for the purpose of eliminating PD-L1^+^ tumor cells. The combination of Vγ2 × PD-L1 with adoptive metastatic Vγ2Vδ2 T cells has been demonstrated to impede the progression of established tumor xenografts and enhance the infiltration of Vγ2Vδ2 T cells into the TME [92]. Another EGFR-Vδ2 bispecific T-cell engager (bsTCE) can activate Vγ9Vδ2 T cells in the PB and tumor samples of EGFR^+^ cancer patients. These T cells express minimal levels of PD-1, TIM3 and LAG3, which in turn mediate the lysis of various tumor samples from EGFR^+^ cancer patients. Significant tumor growth inhibition and improved survival rates were observed in a xenograft mouse model using PBMCs as effector cells [93].

Furthermore, the MHC independent manner of γδ T cells makes them suitable for CAR manipulation. Neuroblastoma mediates immune evasion of Vδ2 T cells by blocking the NKG2D/DAP10 signaling pathway. Therefore, researchers have designed GD2-DAP10-CAR-γδ T cells, which consist of an outer domain that specifically targets GD2, a widely expressed tumor-associated antigen and an inner domain that supports the DAP10 co-stimulatory pathway. CAR-T cells can induce cytotoxicity and decrease PD-1 and TIM3 expression [94]. The co-expression of HLA-G and PD-L1, or the upregulation of PD-L1, may reduce the effectiveness of HLA-G-CAR after adoptive immunotherapy. Concurrent targeting of HLA-G and PD-L1 using a multi-specific CAR will be a suitable solution. The combination of Nb-CAR-γδ T cells and atezolizumab can restore the killing effect of Nb-CAR-γδ T cells and inhibit the growth of 231-R3 tumors in mice, thus prolonging the survival rate of tumor-bearing mice [95]. Simultaneously, using Vδ2 T cells as effector cells, engineered HLA-G-CAR-T cells can secrete the PD-L1/CD3ε bispecific T‐cell engager (BiTE) construct (Nb-CAR. BiTE), which can overcome the limitation of HLA-G and PD-L1, even against tumor cells with inadequate antigen expression, thereby generating potent antitumor effects without obvious toxicity [95].

## Conclusion

The potent cytotoxicity of γδ T cells to tumor cells has garnered growing interest. This review seeks to elucidate their functional status within the TME and establish a foundation for enhancing their therapeutic efficacy. Overall, the expression of exhausted molecules in the tumor immune microenvironment is upregulated, accompanied by changes in the expression of other molecules. However, the upregulation of exhausted molecules does not necessarily indicate a weakened function, but may also indicate adaptive regulation in response to the TME. Moreover, different γδ T cell subtypes may also exhibit different phenotypic changes, but little is known about their functional mechanisms. Understanding their functions and combining them with immune checkpoint therapy could have a significant impact on future immunotherapy. Research has demonstrated that immunosuppressive molecules expressed by γδ T cells can regulate tumor growth and cytokine secretion. Therefore, the impact of γδ T cells on cancer is also limited by PD-1/PD-L1 signaling. Therefore, PD-1/PD-L1 blockade confers benefits not only for αβ T cells but also for γδ T cells; Checkpoint inhibitors can also relieve the immunosuppressive effect of γδ T cells on other cells in the TME, and their cooperation can expand the range of cancer patients who can benefit from immunotherapy [22]. Furthermore, γδ T cells exhibited minimal expression of PD-1 following in vitro expansion, and this low level of PD-1 expression was sustained even after their transfer to NSG mice after tumor transplantation. Consequently, γδ T cells may be less susceptible to inhibitory ligands expressed on tumor cells than endogenous T cells [19]. Therefore, combining immune checkpoint therapy with γδ T cell adoptive transfer therapy will open up a new therapeutic approach.

## Data Availability

Not applicable.

## Abbreviations

ADCC: Antibody dependent cellular cytotoxicity
AML: Acute myeloid leukemia
AREG: Amphiregulin
Arg1: Arginase 1
BATF: Basic leucine zipper transcriptional factor ATF-like
BCG: Bacille Calmette-Guérin
BM: Bone marrows
BMSC: BM-derived stromal cells
bsAb: Bispecific antibody
bsTCE: Bispecific T-cell engager
BTLA: B lymphocyte and T lymphocyte attenuators
BTN: Butylphilic protein
CLM: Colon liver metastatic
CRC: Colorectal cancer
dMMR: DNA mismatch repair-deficient
EOMEs: Eomesodermin
FL: Follicular lymphoma
GZMB: Granzyme B
HCC: Hepatocellular carcinoma
HDAC: Histone deacetylases
HL: Hodgkin lymphoma
HLA: Human leukocyte antigen
HMBPP: Hydroxy-3-methyl-but-2-enyl-pyrophosphate
HVEM: Herpesvirus entry mediator
ICB: Immune checkpoint blockade
ICI: Immune checkpoint inhibitors
ICP: Immune checkpoint
IFN-γ: Interferon γ
IPP: Isopentenyl pyrophosphate
ITSM: Immunoreceptor tyrosine-based switch motif
KIRs: Killer cell immunoglobulin-like receptors
MAI: Mucosal-associated invariant
MALs: Malignant ascites lymphocytes
MCA: Methylcholanthrene
MCPs: Metabolic checkpoints
MDSCs: Myeloid suppressor cells
MGUS: Monoclonal gammopathy of undetermined significance
MM: Multiple myeloma
NKRs: Natural killer cell receptors
NSCLC: Non-small cell lung cancer
OC: Ovarian cancer
OS: Overall survival
pAgs: Phosphoantigens
PB: Peripheral blood
PDAC: Pancreatic ductal adenocarcinoma
PRF1: Perforin
PT: Peripheral tissues
RCC: Renal cell carcinoma
SLAM: Signalling lymphocytic activation molecule
TCGA: The Cancer Genome Atlas
TCRs: T cell receptors
TEXs: Tumor derived exosomes
TILs: Tumor-infiltrating lymphocytes
TLR2: Toll-like receptor 2 ligands
TME: Tumor microenvironment
TNBC: Triple negative breast cancer
TNF: Tumor necrosis factor
Tregs: Regulatory T cells
VDR: Vitamin D receptor
VEGFR2-TKI: Vascular endothelial growth factor receptor tyrosine kinase inhibitor
Zol: Zoledronic acid
